# Association between the severity of hearing loss and the risk of dementia within the 2010–2017 national insurance service survey in South Korea

**DOI:** 10.1038/s41598-020-77752-1

**Published:** 2020-11-26

**Authors:** Young-Soo Chang, Yoon Chan Rah, Min Kyu Lee, Seongbin Park, Bongseong Kim, Kyungdo Han, June Choi

**Affiliations:** 1 Department of Otorhinolaryngology - Head and Neck Surgery, Korea University College of Medicine, Korea University Ansan Hospital, 123, Jeokgeum-ro, Danwon-gu, Ansan-si, Gyeonggi-do 15355 Republic of Korea; 2 Department of Statistics and Actuarial Science, Soongsil University, 369 Sangdo-Ro, Dongjak-Gu, Seoul, 06978 Republic of Korea

**Keywords:** Diseases, Health care, Medical research

## Abstract

Hearing loss and dementia are highly prevalent neurologic conditions in older adults that can considerably impact the quality of life and create social and familial burdens. To investigate the impact of hearing loss on the risk of developing dementia in a nationwide long-term follow-up study using data obtained from the South Korean National Health Information Database. Retrospective medical data for patients of all ages were extracted from the database between January 2010 and December 2017. According to the national disability registry, the degree of severe-profound hearing loss is classified into six grades. We categorized hearing loss into three groups based on the disability registry severity: (1) severe hearing disability (HD), defined as 1st to 3rd grade disabling hearing loss; (2) non-severe HD, 4th and 5th grade disabling hearing loss; and (3) ipsilateral HD, 6th grade disabling hearing loss. After adjusting for potential confounding variables, the hazard ratio (HR) for all dementia types was 1.336 (95% CI 1.306–1.367) in the severe HD group, 1.312 (95% CI 1.286–1.338) in the non-severe HD group, and 1.257 (95% CI 1.217–1.299) in the ipsilateral HD group. On assessing by the age group, the risk of all dementia types in patients younger than 65 years was as follows: HR 1.933 (95% CI 1.779–2.101), 1.880 (95% CI 1.732–2.041), and 1.601 (95% CI 1.435–1.787) in the severe, non-severe, and ipsilateral HD groups, respectively. This study demonstrates that the impact of hearing loss on dementia incidence is severity-dependent, and the risk increases in patients younger than 65 years of age.

## Introduction

Hearing loss (HL) and dementia are both highly prevalent neurologic conditions in older adults and can have a considerable impact on patients’ quality of life and contribute to social and familial burdens^1,2^.

Proper interventions to delay dementia onset by 1 year would decrease the global prevalence of dementia by more than 10% by 2050^3^; however, there are no known interventions that are currently effective in minimizing the burden of this disease.

After Lin et al. first described the association between HL and dementia^4^, a growing body of literature has suggested that the two conditions are interrelated and that HL may be an independent risk factor for the development of dementia in older adults^5^. Although several epidemiological studies have demonstrated this association^6–8^, the causal link of how HL increases the risk of developing dementia is not well understood. To investigate this causal link, a prospective, large-scale, long-term follow-up study would be beneficial.

Recently, several nationwide studies with large-scale, long-term follow-up designs have been published from South Korea^9,10^. South Korea has several advantages for conducting a nationwide study that addresses the association between HL and dementia risk: the territory is relatively small; it benefits from excellent transportation to accessible medical services, and the national population is registered with the National Health Insurance Service, with all medical data being well-organized in their respective National Health Information Database (NHID). Thus, the analysis of this nationwide data may provide a deeper understanding of this association.

Therefore, the purpose of this study was to investigate the impact of HL on the risk of developing dementia using nationwide long-term follow-up data obtained from the NHID in South Korea.

## Materials and methods

### Study design and participants

This study used nationwide data from the NHID, which is operated by the Korean National Health Insurance Service (KNHIS). The KNHIS is a government-affiliated agency under the Korean Ministry of Health and Welfare that supervises all medical activities in Korea. All Korean citizens and registered foreigners are enrolled in and receive medical services from the KNHIS^9^. We also used nationwide data from the National Disability Registry (NDR), a social registry, which provides patients with severe-profound HL or deafness with disability benefits such as financial assistance to acquire hearing aids or cochlear implants^9^.

**Figure 1:**
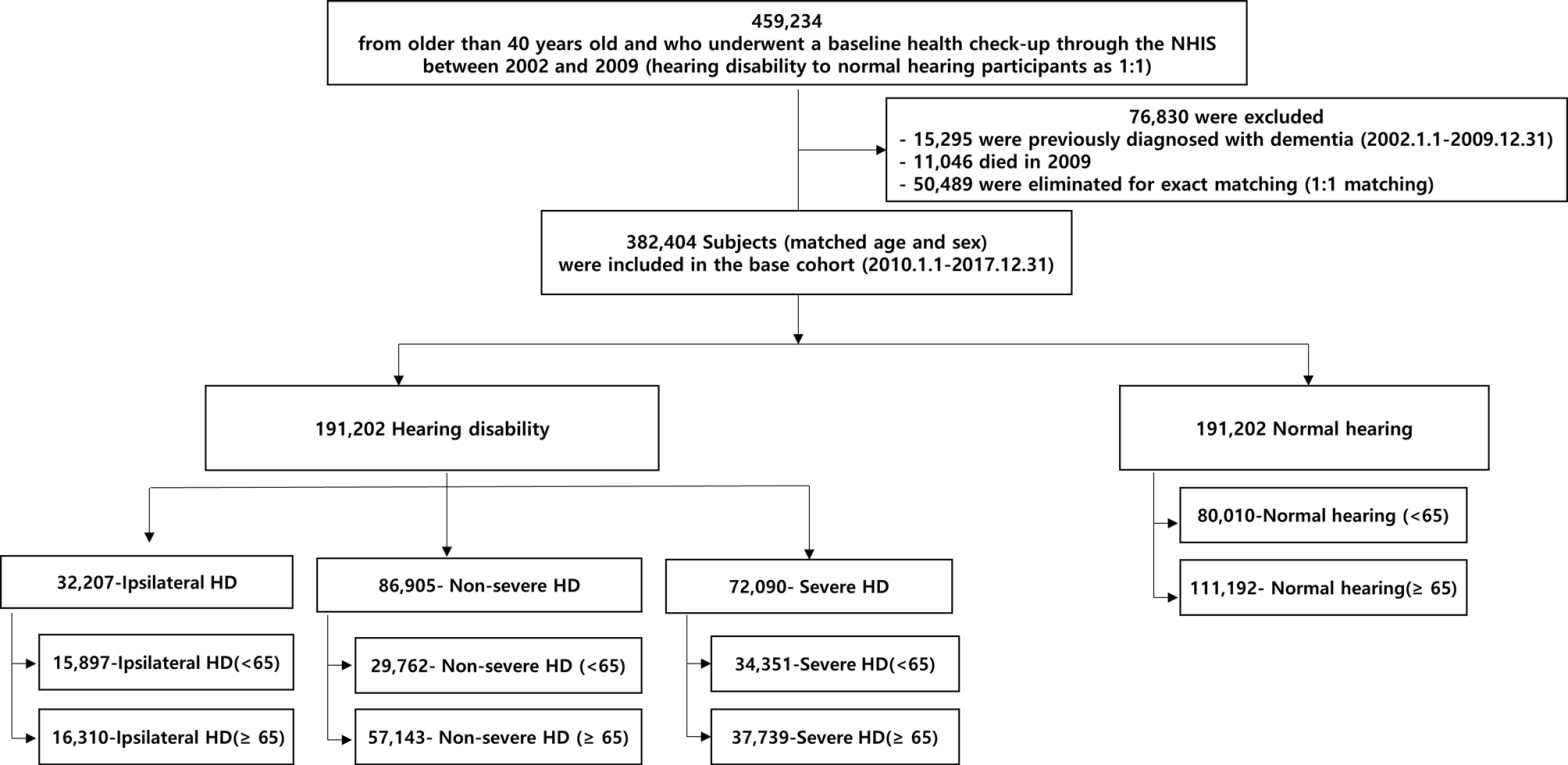
Study flow chart.

We initially recruited 459,234 participants who were older than 40 years and who underwent a baseline health checkup through the NHIS between 2002 and 2009. The ratio of hearing disabled to normal hearing participants was 1:1. From this population, we excluded 76,830 participants who were previously diagnosed with dementia (15,295), had died in 2009 (11,046), or were eliminated for exact matching (50,489). Ultimately, 382,404 age- and sex-matched participants were included in the baseline cohort to be analysed; this included 191,202 participants registered as hearing disabled and a matched pool of 191,202 participants with normal hearing (Fig. 1).

Retrospective medical data for patients of all ages were extracted from the NHID between January 2010 and December 2017. These data did not involve identifiable personal data such as the patient’s name but did provide the age, sex, number of patients, and the national classification code of disease. Therefore, the KNHIS approved our nationwide study without the need to obtain informed consent from patients^9^.

The NHID and NDR contained information on the demographics of each patient, degree of HL, medical service use, medication, deductions, and claims. According to the NDR, the degree of severe-profound HL was classified into six grades: 1st grade disabling HL (both-side HL ≥ 90 dB HL and speech disorder), 2nd grade (both-side HL ≥ 90 dB HL), 3rd grade (both-side HL ≥ 80 dB HL), 4th grade (both-side HL ≥ 70 dB HL), 5th grade (both-side HL ≥ 60 dB HL), and 6th grade (worse-side HL ≥ 80 dB HL and other-side HL ≥ 40 dB HL)^9^. In this study, we categorized HL into three groups based on the severity of the NDR degree: (1) severe hearing disability (HD), defined as 1st to 3rd grade disabling HL; (2) non-severe HD, defined as 4th and 5th grade disabling HL; and (3) ipsilateral HD, defined as 6th grade disabling HL.

Pure-tone thresholds were obtained using the pure-tone averages (PTAs) at four frequencies (0.5 kHz + 2 × 1 kHz + 2 × 2 kHz + 4 kHz)/6). To be registered in the NDR as having severe-profound HL, patients need to undergo PTA tests at least three times within an interval of 3–7 days, and the PTA test results must be confirmed using the auditory brainstem response or auditory steady-state response. The KNHIS reviews the hearing test results and confirms registration to the NDR, while otologists review and debate over issues concerning the hearing test results^9^. Consequently, the analysis of the NDR can yield reliable information about the true prevalence of severe-profound HL in South Korea^9^.

### Data collection

We collected the following baseline data from the NHIS database: age (years), sex, income level (lowest 20%), and patient data on comorbidities including diabetes, hypertension, dyslipidaemia, chronic obstructive pulmonary disease, ischemic heart disease, stroke, chronic heart failure, atrial fibrillation, cancer, and end-stage renal disease. To analyse the risk of dementia, patient data concerning dementia, including Alzheimer’s dementia (AD), vascular dementia (VD), and other causes of dementia (OD), were also collected. Dementia patients were defined as those who were prescribed more than one drug (donepezil, rivastigmine, galantamine, and memantine) for the International Classification of Diseases, 10th edition (ICD-10) codes (AD: F00 or G30; VD: F01; OD: F02, F03, or G31). When the patients had codes for both AD and VD, the main diagnosis was defined as the main code. If codes for both AD and VD were assigned as an additional diagnosis, the main diagnosis was defined as the main code at the next visit. If AD or VD was not defined as the main diagnosis until the second claims database, the main diagnosis was considered as OD^11^.

### Statistical analysis

Data are presented as the mean ± standard deviation (SD) for age and as proportions for the remaining categorical variables. Statistical analysis was performed using SAS software, version 9.3 (SAS Institute, Cary, North Carolina, USA). However, this study used data obtained from the entire population of South Korea; therefore, no data on SD were available, and an analysis to predict universal population trends was not required. All data generated or analysed in this study are available from the corresponding author upon reasonable request. Baseline characteristics of study participants were compared according to the severity of HL using the ANOVA for continuous variables and the chi-squared test for categorical variables. The incidence rate (IR) of dementia was analysed using Kaplan–Meier estimates and compared according to HL severity. In addition, to assess the significance of dementia risk after adjusting for possible confounding variables, Cox proportional hazard models were used to estimate the hazard ratio (HR) and the corresponding 95% confidence interval (CI) for the association between dementia and HL. Potential confounders included in the adjustment were age, sex, income, diabetes, hypertension, dyslipidaemia, chronic obstructive pulmonary disease, ischemic heart disease, stroke, chronic heart failure, atrial fibrillation, cancer, and end-stage renal disease.

### Ethical approval

The study was approved and exempted by the institutional review board of the Korea University College of Medicine (KUMC IRB 2019AS0029 & 2020AS0292) because of the use of publicly available data. All methods were performed in accordance with relevant guidelines and regulations.

## Results

A total of 191,202 participants were registered in the NDR in 2009. Of these, 72,090 participants were classified with 1st grade to 3rd grade disabling HL, and 86,905 participants were classified with 4th and 5th grade disabling HL. The number of participants with 6th grade disabling HL, defined as those with worse-side HL ≥ 80 dB HL and other-side HL ≥ 40 dB HL, was 32,207. In the age- and sex-matched group, 191,202 normal hearing participants were identified and enrolled in the study. The follow-up duration was 6.97 ± 2.1 years for normal hearing, 7.04 ± 2.04 years for ipsilateral HD, 6.58 ± 2.35 years for non-severe HD, and 6.79 ± 2.24 years for severe HD. Table 1 details the raw data and associated percentages of the total population.

**Table 1:** Baseline characteristics according to the severity of hearing loss.

n	Normal	Ipsilateral HD	Non-severe HD	Severe HD	*p* value
	191,202	32,207	86,905	72,090	
**Age (years)**	65.91 ± 11.47	63.86 ± 10.95	68.04 ± 10.95	64.24 ± 11.85	< .0001
≥ 65 (%)	111,192 (58.15)	16,310 (50.64)	57,143 (65.75)	37,739 (52.35)	< .0001
Sex, male (%)	10,1093 (52.87)	18,173 (56.43)	44,858 (51.62)	38,062 (52.8)	< .0001
Income, lowest 20% (%)	38,977 (20.39)	8277 (25.70)	23,218 (26.72)	24,286 (33.69)	< .0001
Diabetes (%)	25,548 (13.36)	4765 (14.79)	13,335 (15.34)	8919 (12.37)	< .0001
Hypertension (%)	80,147 (41.92)	13,248 (41.13)	39,874 (45.88)	27,600 (38.29)	< .0001
Dyslipidaemia (%)	30,870 (16.15)	5621 (17.45)	14,624 (16.83)	9696 (13.45)	< .0001
COPD (%)	21,210 (11.09)	4347 (13.50)	13,406 (15.43)	9628 (13.36)	< .0001
IHD (%)	20,945 (10.95)	3851 (11.96)	11,450 (13.18)	7390 (10.25)	< .0001
Stroke (%)	9631 (5.04)	1805 (5.6)	6169 (7.10)	4200 (5.83)	< .0001
CHF (%)	4597 (2.40)	790 (2.45)	3045 (3.50)	2030 (2.82)	< .0001
AF (%)	3088 (1.62)	546 (1.70)	1787 (2.06)	1173 (1.63)	< .0001
ESRD (%)	71 (0.04)	23 (0.07)	86 (0.10)	243 (0.34)	< .0001
Cancer (%)	7240 (3.79)	1363 (4.23)	3665 (4.22)	2432 (3.37)	< .0001
**Outcome**					
Dementia (%)	24,326 (12.72)	4254 (13.21)	16,275 (18.73)	10,435 (14.47)	< .0001
AD (%)	18,558 (9.71)	3140 (9.75)	12,288 (14.14)	7904 (10.96)	< .0001
VD (%)	2596 (1.36)	499 (1.55)	1781 (2.05)	1145 (1.59)	< .0001
OD (%)	3172 (1.66)	615 (1.91)	2206 (2.54)	1386 (1.92)	< .0001
F/U duration (year)	6.97 ± 2.1	7.04 ± 2.04	6.58 ± 2.35	6.79 ± 2.24	< .0001

HD, hearing disability; COPD, chronic obstructive pulmonary disease; IHD, ischemic heart disease; CRF, chronic heart failure; AF, atrial fibrillation; ESRD, end-stage renal disease; AD, Alzheimer’s dementia; VD, vascular dementia; OD, other causes of dementia; F/U, follow-up.

**Figure 2:**
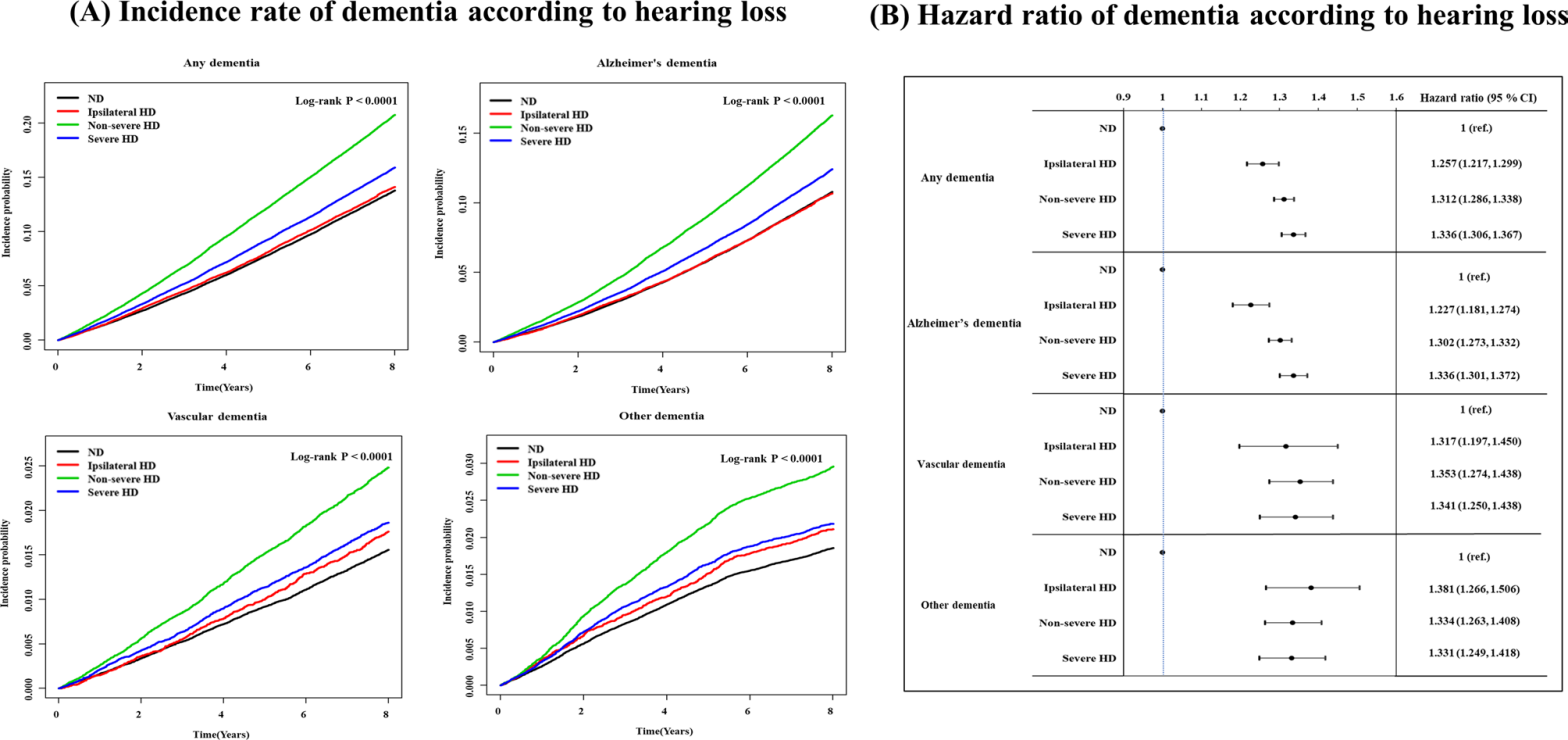
Incidence rate and hazard ratio according to hearing loss. After adjusting for potential associated factors, the incidence rate (**A**) and hazard ratio (**B**) of dementia was higher in the hearing loss group than in the normal hearing group. ND, non-disability; HD, hearing disability.

In Kaplan–Meier analysis, increases in the IR of dementia (log-rank test *p* < 0.0001) were frequently observed in patients with HL. During the follow-up period, 10,435 cases of any dementia type (IR = 21.318 per 1000 person-years) were observed in participants with severe HD; 16,275 cases of any dementia type (IR = 28.459 per 1000 person-years) were observed in participants with non-severe HD; and 4254 cases of any dementia type (IR = 18.767 per 1000 person-years) were observed in participants with ipsilateral HD. In contrast, 24,326 cases of any dementia type (IR = 18.265 per 1000 person-years) were observed in the normal hearing group. In addition, after adjusting for potential confounding variables, the HR for all dementia types was 1.336 (95% CI 1.306–1.367) in the severe HD group, 1.312 (95% CI 1.286–1.338) in the non-severe HD group, and 1.257 (95% CI 1.217–1.299) in the ipsilateral HD group. HRs for Alzheimer’s dementia were 1.336 (95% CI 1.301–1.372) in the severe HD group, 1.302 (95% CI 1.273–1.332) in the non-severe HD group, and 1.227 (95% CI 1.181–1.274) in the ipsilateral HD group. For risk of vascular dementia, the HR was 1.341 (95% CI 1.250–1.438) in the severe HD group, 1.353 (95% CI 1.274–1.438) in the non-severe HD group, and 1.317 (95% CI 1.197–1.450) in the ipsilateral HD group. For the risk of other causes of dementia the HR was 1.331 (95% CI 1.249–1.418), 1.334 (95% CI 1.263–1.408), and 1.381 (95% CI 1.266–1.506) for the severe, non-severe, and ipsilateral HD groups, respectively (Fig. 2, Table 2). All *p* values were < 0.0001.

**Table 2:** The risk of dementia type according to the severity of hearing loss.

	Number	Dementia	Duration (year)	IR (per 1000)	Hazard ratio (95% CI)	*p* value for trend
**Any dementia**						
ND	191,202	24,326	1,331,848.69	18.265	1 (ref.)	< .0001
Ipsilateral HD	32,207	4254	226,679.63	18.767	1.257 (1.217, 1.299)	
Non-severe HD	86,905	16,275	571,866.43	28.459	1.312 (1.286, 1.338)	
Severe HD	72,090	10,435	489,501.1	21.318	1.336 (1.306, 1.367)	
**Alzheimer’s dementia**						
ND	191,202	18,558	1,331,848.69	13.93	1 (ref.)	< .0001
Ipsilateral HD	32,207	3140	226,679.63	13.852	1.227 (1.181, 1.274)	
Non-severe HD	86,905	12,288	571,866.43	21.488	1.302 (1.273, 1.332)	
Severe HD	72,090	7904	489,501.1	16.147	1.336 (1.301, 1.372)	
**Vascular dementia**						
ND	191,202	2596	1,331,848.69	1.950	1 (ref.)	< .0001
Ipsilateral HD	32,207	499	226,679.63	2.201	1.317 (1.197, 1.450)	
Non-severe HD	86,905	1781	571,866.43	3.114	1.353 (1.274, 1.438)	
Severe HD	72,090	1145	489,501.1	2.339	1.341 (1.250, 1.438)	
**Other dementia**						
ND	191,202	3172	1,331,848.69	2.382	1 (ref.)	< .0001
Ipsilateral HD	32,207	615	226,679.63	2.713	1.381 (1.266, 1.506)	
Non-severe HD	86,905	2206	571,866.43	3.858	1.334 (1.263, 1.408)	
Severe HD	72,090	1386	489,501.1	2.831	1.331 (1.249, 1.418)	

ND, non-disability; HD, hearing disability; IR, incidence rate; CI, confidence interval. Duration (years): sum of the observation period.

On assessing the incidence of dementia by the age group, the risk of all dementia types in those younger than 65 years was an HR of 1.933 (95% CI 1.779–2.101), 1.880 (95% CI 1.732–2.041), and 1.601 (95% CI 1.435–1.787) in the severe, non-severe, and ipsilateral HD groups, respectively. All *p* values were < 0.0001. The risk of Alzheimer’s dementia in those younger than 65 years showed similar trends to that of all dementia types. However, the risk of vascular dementia differed (HR = 1.938 [95% CI 1.578–2.380] in the severe HD group; HR = 1.979 [95% CI 1.618–2.421] in the non-severe HD group; and HR = 1.732 [95% CI 1.331–2.254] in the ipsilateral HD group; *p* < 0.0001). The risk of dementia, including Alzheimer’s dementia, vascular dementia, and other causes of dementia in adults older than 65 years was similar across the HD groups (Table 3).

**Table 3:** The risk of each type of dementia according to patient age.

Hearing group	Number	Dementia	Duration (year)	IR (per 1000)	Hazard ratio (95% CI)	*p* value for trend
**Any dementia**						
Age (< 65)						
ND	80,010	1278	623,634.39	2.049	1 (ref.)	< .0001
Ipsilateral HD	15,897	430	122,763.98	3.503	1.601 (1.435, 1.787)	
Non-severe HD	29,762	1049	228,157.65	4.598	1.880 (1.732, 2.041)	
Severe HD	34,351	1042	262,881.64	3.964	1.933 (1.779, 2.101)	
Age (≥ 65)						
ND	111,192	23,048	708,214.30	32.544	1 (ref.)	< .0001
Ipsilateral HD	16,310	3824	103,915.64	36.799	1.231 (1.19, 1.274)	
Non-severe HD	57,143	15,226	343,708.78	44.299	1.281 (1.255, 1.308)	
Severe HD	37,739	9393	226,619.47	41.448	1.300 (1.269, 1.332)	
**Alzheimer’s dementia**						
Age (< 65)						
ND	80,010	884	623,634.39	1.418	1 (ref.)	< .0001
Ipsilateral HD	15,897	284	122,763.98	2.313	1.532 (1.339, 1.751)	
Non-severe HD	29,762	732	228,157.65	3.208	1.891 (1.714, 2.087)	
Severe HD	34,351	745	262,881.64	2.834	2.012 (1.822, 2.221)	
Age (≥ 65)						
ND	111,192	17,674	708,214.30	24.956	1 (ref.)	< .0001
Ipsilateral HD	16,310	2856	103,915.64	27.484	1.207 (1.16, 1.256)	
Non-severe HD	57,143	11,556	343,708.78	33.622	1.273 (1.244, 1.304)	
Severe HD	37,739	7159	226,619.47	31.590	1.300 (1.264, 1.336)	
**Vascular dementia**						
Age (< 65)						
ND	80,010	208	623,634.39	0.334	1 (ref.)	< .0001
Ipsilateral HD	15,897	76	122,763.98	0.619	1.732 (1.331, 2.254)	
Non-severe HD	29,762	177	228,157.65	0.776	1.979 (1.618, 2.421)	
Severe HD	34,351	173	262,881.64	0.658	1.938 (1.578, 2.380)	
Age (≥ 65)						
ND	111,192	2388	708,214.30	3.372	1 (ref.)	< .0001
Ipsilateral HD	16,310	423	103,915.64	4.071	1.269 (1.144, 1.408)	
Non-severe HD	57,143	1604	343,708.78	4.667	1.301 (1.221, 1.386)	
Severe HD	37,739	972	226,619.47	4.290	1.281 (1.188, 1.38)	
**Other dementia**						
Age (< 65)						
ND	80,010	186	623,634.39	0.298	1 (ref.)	< .0001
Ipsilateral HD	15,897	70	122,763.98	0.5702	1.783 (1.354, 2.35)	
Non-severe HD	29,762	140	228,157.65	0.614	1.721 (1.38, 2.145)	
Severe HD	34,351	124	262,881.64	0.472	1.557 (1.236, 1.961)	
Age (≥ 65)						
ND	111,192	2986	708,214.30	4.216	1 (ref.)	< .0001
Ipsilateral HD	16,310	545	103,915.64	5.245	1.342 (1.225, 1.471)	
Non-severe HD	57,143	2066	343,708.78	6.010	1.310 (1.238, 1.386)	
Severe HD	37,739	1262	226,619.47	5.569	1.318 (1.234, 1.408)	

ND, non-disability; HD, hearing disability; IR, incidence rate; CI, confidence interval. Duration (years): Sum of the observation period.

## Discussion

After Lin et al. reported that HL is independently associated with incident all-cause dementia, the role of HL in developing dementia has been heavily explored^4^. In the present large-scale national data analysis, we present results consistent with those of previous studies^4,6,7^.

In this study, we used the nationwide data from the NDR to obtain information on the degree of HL and from the KNHIS with all medical data being well-organized in their respective NHID.

All Korean citizens are enrolled in the KNHIS. HD patients registered with the NDR were classified depending on the severity of hearing loss.

Recently, several nationwide studies have reported different databases on dementia registration for each country. For example, the Swedish dementia registry (SveDem) is registered by the date when dementia diagnosis is established^12^. In Czech Republic, data of patients hospitalized for dementia are provided by the national register of hospitalized patients in all Czech hospitals^13^. In New South Wales (NSW), Australia, recruitment to the 45 and up study involves patients diagnosed with dementia from 2006 to 2009 through random sampling from the Department of Human Services enrollment database (Australia’s national universal insurance provider)^14^. Although the ICD-10 codes for dementia and additional details for defining dementia, such as medications for treating dementia, hospital admissions, aged care assessments, and underlying or contributing cause of death, are slightly different for each study, our definition for dementia is almost similar to that of previous studies^12–16^.

We also used nationwide data from the National Disability Registry (NDR), a social registry, which provides patients with severe-profound HL or deafness with disability benefits such as financial assistance to acquire hearing aids or cochlear implants.

Our results reveal that the association between HL and the risk of dementia is severity-dependent. The risk of any dementia type in participants with severe HD increased to 1.336 when compared to participants with normal hearing. Similarly, the risk of Alzheimer’s dementia in participants with severe HD also increased to 1.336 with normal hearing participants as the reference group. This association remained despite controlling for confounders of age, sex, income level, and the comorbidities of diabetes, hypertension, dyslipidaemia, chronic obstructive pulmonary disease, ischemic heart disease, stroke, chronic heart failure, atrial fibrillation, cancer, and end-stage renal disease.

Furthermore, evaluation of the ipsilateral HD group showed a relatively low risk of dementia in patients with some degree of hearing preservation. The risk of dementia and Alzheimer’s dementia was 1.257 and 1.227, respectively, and these values were statistically lower than those of the severe HD group (both HR = 1.336). These results show that severe ipsilateral HL (worse-side HL ≥ 80 dB HL) with better contralateral HL (better-side HL ≥ 40 dB HL) contributes to the development of any dementia type and Alzheimer’s dementia. However, the extent of this contribution of ipsilateral HD might be weaker than that of severe HD. It can therefore be estimated that the risk of dementia decreases when the effect of HL on dementia is limited due to better hearing ipsilaterally (current better-side HL ≥ 40 dB HL). Based on these estimates, it is conceivable that patients with HL should have some level of hearing threshold via rehabilitation such as hearing aids or cochlear implants. Nevertheless, considering that few studies have examined the role of hearing interventions, our results highlight the need for further studies to assess the clinical benefit of interventions that acquire better hearing capacity on one side for patients with severe HD.

In contrast, the severity-dependent association between HL and the risk of dementia does not hold when considering the risk of vascular dementia. One possible explanation is that having a stroke places people at high risk of dementia in the long term, with approximately 20–25% of subjects developing delayed dementia^17^, and vascular dementia is closely associated with vascular risk factors and cerebrovascular disease^18^. This implies that vascular dementia may possess a nature that is somewhat different from other dementia types in terms of disease progression.

Furthermore, the prevalence of dementia is increasing alongside its socioeconomic burden. Prevention is the best method to reduce the socioeconomic costs of dementia. Recently, several studies have investigated the causes of dementia, with HL identified as one of the strongest risk factors for developing dementia^4,6^. One possible explanation for this association is the impact of HL on cortical processing. HL increases cognitive load, diverting cognitive resources to auditory processing at the expense of other cognitive processes such as working memory^19,20^. As age-related HL is accompanied by auditory cortex atrophies^21,22^, adults with HL may be difficult to recruit different neural resources for speech comprehension; older adults may rely on additional resources such as the middle frontal gyrus and a core speech comprehension network normally recruited by younger adults, suggestive of a compensatory mechanism.

Social isolation due to HL could be another reason for the development of dementia^23,24^. In one study with a 4-year follow-up assessment of 6034 participants (mean age at baseline = 65.6 years), loneliness and isolation were associated with poorer cognitive function among older adults^23^. As noted previously, one of the mechanisms by which social integration aids cognitive function is through communication and the interaction necessitated by social engagement as well as by the sense of worth and affiliation provided by fulfilling contacts^24,25^. Additionally, both HL and dementia share a similar pathophysiology, and various environmental factors contribute to developing both HL and dementia^8,26^. Several studies have found HL to be associated with dementia or cognitive decline. Together, this body of evidence substantially supports the hypothesis that HL is a risk factor for dementia. Because of the considerable variability among existing studies regarding how dementia, HL, and potential confounding variables were determined, careful scrutiny and comparison of these methods are crucial to confirm the validity of this observed association.

In addition, applying the knowledge gained from these results allows us to advocate the clinical importance of HL management in patients younger than 65 years of age. In the present study, we assessed the risk of dementia according to age group and found that patients with HL younger than 65 years of age showed a significantly different risk of dementia according to HL severity. This is a novel finding that may be explained by other exposures to other risk factors for dementia, such as occurrence of HL earlier in life. In younger patients, it may be inferred that HL is the key risk factor associated with dementia, as opposed to vascular risk factors or cerebrovascular disease.

One notable limitation of the present study is that we were unable to assess the impact of hearing aid or cochlear implant use on dementia, as some of the enrolled participants might have used hearing aids or cochlear implants due to HL. Although the role of hearing aids or cochlear implants in preventing the development of dementia is still controversial^27–30^, this association could have been investigated through further analysis. Additional prospective studies with reliable methods for determining hearing aid or cochlear implant use and compliance are necessary to fully ascertain the impact of hearing aid on cognitive decline. In addition, the inherent limitations of a large-scale retrospective study design may have influenced our results. Based on the present study, it can be advocated that HL is a risk factor for incident dementia.

In conclusion, we used nationwide long-term follow-up data to demonstrate that the impact of HL on the risk of developing dementia in the South Korean population is severity-dependent. This risk is higher in patients younger than 65 years of age.
